# Bioactive glass in the treatment of chronic osteomyelitis—a valid option?

**DOI:** 10.1097/OI9.0000000000000105

**Published:** 2021-06-15

**Authors:** Franziska Ziegenhain, Valentin Neuhaus, Hans-Christoph Pape

**Affiliations:** Department of Trauma, University Hospital Zurich, Zurich, Switzerland

**Keywords:** bioglass treatment, chronic infection, osteomyelitis, polymethyl methacrylate cement, S53P4

## Abstract

Chronic osteomyelitis continues to represent a challenge both for patients and the treating physician, especially in the presence of multiple germs. We performed a literature review assessing the current role of the indications of bioactive glass. We included studies about patients with chronic osteomyelitis that were treated with S53P4. A literature review of Medline via PubMed was performed. After the exclusion of case reports, 7 studies were included in the narrative review. Recurrence of infection was defined as the main outcome parameter. Six of 7 studies were retrospective, or case studies with a relatively small sample size (total patient number N = 274). The overall recurrence rate was 10.6%. Studies that compared the outcome of the treatment with S53P4 versus antibiotic-loaded polymethyl methacrylate (PMMA) revealed no significant difference. The data reviewed indicate that in cases of multiple bacteria and resistance to antibiotic treatment, bioglass may be a valuable treatment alternative to other forms of spacers (e.g., PMMA). This statement is limited by the fact that the number of described cases is very low. Therefore, a definitive statement of its effectiveness cannot be made at this point.

## 1 Introduction

Bacterial osseous infections occur after trauma, surgery, or via the bloodstream pathways.^[1,2]^ Following open fractures, a chronic infection represents a special threat.^[3]^ Formation of sequestra in the bone with consequent loss of stability, lack of fracture healing, and significant pain and restrictions may occur.^[2]^

Different approaches for treatment have been established,^[4,5]^ among which systemic antibiotic therapy is one of the cornerstones.^[6,7]^ In addition, surgical intervention to eradicate necrotic and infected tissue is crucial and thorough debridement is fundamental. However, this leaves the patient with a large substance defect that needs to be addressed.^[8]^ To support the healing of the bone and provide stability, different methods for filling these dead spaces have been established. The most frequently used option is to fill defects with polymethyl methacrylate (PMMA) loaded with antibiotics, for example, implantation of cement rods for long bones.^[7,9]^ This requires a second surgery to remove the cement and perform a definitive stabilization.^[10,11,12]^

Another option for filling the resulting cavity is bioactive glass. The most frequently used form to date is named S53P4. Its composition (53% SiO_2_, 23% Na_2_O, 20% CaO, and 4% P_2_O) is osteoinductive and increases local pH as well as osmotic pressure, which leads to its antibacterial effect. This is achieved by its biochemical characteristics. Its osteoinductive features are composed of the formation of silica layer precipitation of calcium phosphate on the surface of the bioglass. These structures crystallize into hydroxyapatite that then induces the formation of new bone. This process continues until the bioactive glass is completely absorbed.^[13,14,15,16,17]^

The increase in local pH and osmotic pressure is attained through the release of ions from the bioactive glass. This provides a hostile environment for bacterial growth.^[18,19]^

In this study, we provide an overview of the published work about the clinical outcome of chronic osteomyelitis treated with bioglass.

## 2 Materials and methods

### 2.1 Inclusion criteria

We only included studies that report the outcome after the clinical use of bioactive glass in the treatment of chronic osteomyelitis. Studies that compared the use of bioactive glass to other materials, and studies exclusively focused on the use of bioglass were included. Studies that focused on the treatment of chronic osteomyelitis of the long bones as well as the axial skeleton were also included.

### 2.2 Exclusion criteria

Case studies and in vitro results were excluded. Studies focusing on the use of bioactive glass in the treatment of bone tumors were excluded.

### 2.3 Main outcome measure

The main outcome was defined as the absence of recurrence of infection.

### 2.4 Search and selection process

We searched the Medline Database via PubMed for studies regarding the use of bioactive glass for the treatment of chronic osteomyelitis between January 1, 1990 and May 31, 2020. The search included articles in English, German, and French languages. Reviews and commentaries were excluded. Studies were first sorted by abstracts and title to define if they met the inclusion criteria.

The remaining articles were reviewed for complete content. Duplicates were searched for by comparing authors, sample sizes, and outcome.

## 3 Results

A total of 301 search results were available on PubMed. Seven studies regarding the outcome of the treatment of chronic osteomyelitis could be identified.^[8,20,21,22,23,24]^ These studies investigated the outcome of the clinical use of bioactive glass in the treatment of chronic osteomyelitis. Six of 7 studies were retrospective, or case studies, with a relatively small sample size (total patient number N = 274). An overview of the included studies is given in Table 1.

**Table 1:** Overview of the included studies. The recurrence of infection is listed for the overall patients included in the study as well as for the subgroups N (subgroup A/B).

Author	Study design	Bone substitutes	N	Patients treated with S53P4	Recurrence of infection	Localization	Conclusion
Ferrando et al.	Retrospective single center study	S53P4 vs. antibiotic loaded PMMA Beads	25	12	2 (1/1)	Tibia, Femur, Calcaneus	Bioactive glass and Antibiotic beads are equally effective
McAndrew et al.	Retrospective single center study	S53P4	3	3	0	Tibia, Femur, Ulna	Excellent clinical outcome, no recurrence of infection in three cases
Kankare et al.	Case study	S53P4	3	3	0	Spine	Suitable for severe vertebral osteomyelitis
Lindfors et al.	Multicenter study	S53P4	116	116	12	Tibia, Femur, Calcaneus, Other	90% success Rate in Treatment with S53P4
Geurts et al.	Single center retrospective study	S53P4/Gentamicin loaded PMMA	50	25	7 (2/5)	Tibia, Femur, Humerus, Other	Eradication in 92%, Use of S53P4 is cost-effective
Malat et al.	Single center retrospective study	S53P4	50	50	7	Tibia, Calcaneus, Foot, Others	Good outcome, Thorough Debridement is essential
Drago et al.	Prospective clinical study	S53P4	27	27	3	Tibia, Femur, Humerus, Other	Good clinical outcome

PMMA, polymethyl methacrylate.

### 3.1 Study design

Of the 7 included studies, only 1 was conducted prospectively. All data used in the remaining studies were collected retrospectively. Two of the studies compared the outcome between patients treated with antibiotic-loaded beads and patients treated with bioactive glass.

### 3.2 Patient collective

The sample size ranged between 3 and 116 patients. One hundred ninety-six of the treated patients were male. All the patients were diagnosed with osteomyelitis. Most studies described that most of the patients already had underwent a multitude of surgeries and interventions that were not successful. In all the studies concerning the outcome of the usage of bioactive glass in chronic osteomyelitis of long bones, the tibia was the most frequent affected bone.

### 3.3 Surgical procedure and antibiotic treatment

All studies used S53P4 bioactive glass (BonAlive Biomaterials Ltd., Turku, Finland) without further addition of local antibiotics. The surgical procedure was described similarly for all studies except the study of Kankare et al^[21]^ focusing on vertebral osteomyelitis. In all other cases, a thorough debridement of all necrotic tissue was performed. Fistulas were excised. The bone cavity was debrided, irrigated, and filled with bioactive glass. Primary wound closure was always attempted. In some cases, plastic coverage was needed. All patients received systemic antibiotic therapy according to the microbiological findings.

### 3.4 Outcome

Follow-up was a minimum of 12 months in all studies. Overall, 31 patients (11.3%, N = 274) had recurrence of infections during the time of follow up. Of the patients treated with S53P4 (N = 236), 25 (10.6%) had a relapse. The studies that compared antibiotic-loaded PMMA with S53P4 showed no significant difference in the outcome.^[8,22]^ S53P4 was generally very well tolerated. The clinical outcome (weight bearing, pain, fracture healing) was in all studies described as very good.

## 4 Discussion

Chronic osteomyelitis is addressed by different options of treatment and many of them may be a valid option. For recurrent infection in the treatment of chronic osteomyelitis, several in vitro studies regarding the antibacterial and osteoinductive characteristics of S53P4 have shown effectiveness toward inhibiting bacterial growth, whereas stimulating new bone growth and angiogenesis.^[13,17,18,19,25]^

One of the advantages of bioactive glass has been argued to be its antibacterial effect without local antibiotic treatment.^[6]^ To our knowledge, there is only 1 study available that analyzes the cost-effectiveness of the treatment with bioglass compared to other established osteomyelitis treatments.^[22]^ This study shows a clear trend to lower costs in the use of S53P4. This is noteworthy, as the treatment costs of chronic osteomyelitis are extremely high.

**Figure 1:**
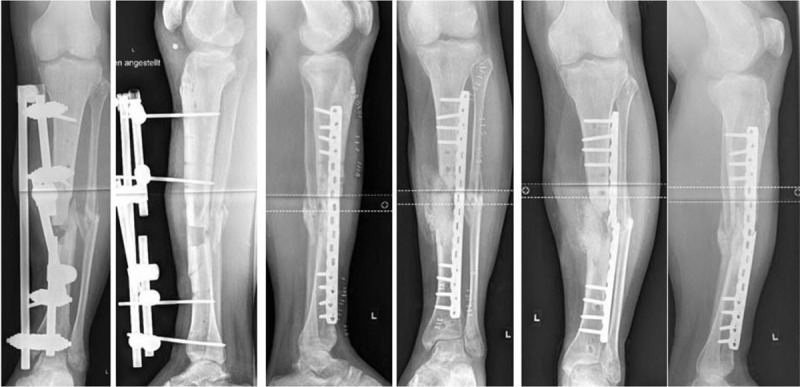
Twenty-five-year-old male with chronic osteomyelitis after second degree open tibia fracture, who underwent multiple revision surgeries. After thorough debridement and filling of the defect with Bioglass and cancellous bone, plate osteosynthesis was performed. Four months later, plate failure occurred as a sign of incomplete ingrowth of the spacer and no signs of fracture consolidation.

The data obtained on the clinical use of bioactive glass for the treatment of chronic osteomyelitis, however, are to this date poor as the number of the cases described is low. Figure 1 shows an example of a treatment failure with subsequent lack of bone healing and failure of osteosynthesis, thus requiring further surgery. Nevertheless, the outcome of these studies obtained over the past few years seems very promising. The comparative studies between the use of S53P4 with PMMA loaded with antibiotics, demonstrated no significant difference in outcome.

**Figure 2:**
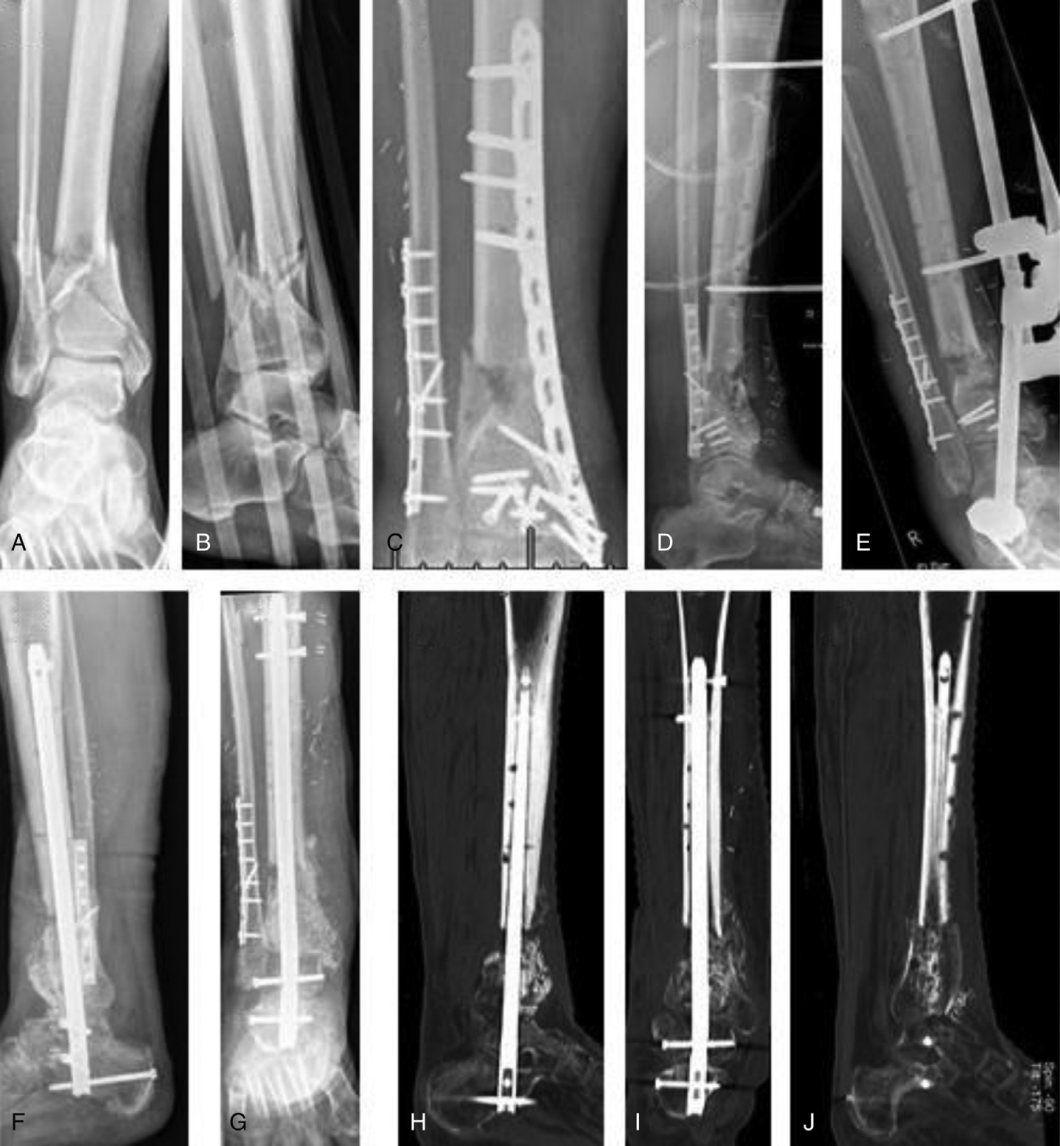
Fifty-nine-year-old male with chronic osteomyelitis after pilon fracture (A, B). Initial treatment with AO external fixator followed by plate osteosynthesis. Ten months after initial treatment, persisting secretion from wound and failure of osteosynthesis was observed (C, D, E). Treatment with debridement, arthrodesis, and bioglass (F, G). Follow-up CT scans show partial consolidation (H, I, J), the patient was pain free and able to bear full weight. CT = computed tomography.

S53P4 is a last resort treatment option for patients with severe cases of chronic osteomyelitis. As shown in Figure 2, the clinical outcome can be satisfactory. In summary, it can be stated that the use of bioactive glass could be considered in cases of osteomyelitis. Thorough surgical debridement and sufficient filling of the cavity seem to be critical for the success.

In conclusion, the use of bioactive glass in the treatment of chronic osteomyelitis has not been investigated in depth. Nevertheless, existing studies show certain promising clinical results, especially if used as a last resort option in recurrent cases.
